# Comparison of patient classification systems for dimensioning nursing staff

**DOI:** 10.1590/1980-220X-REEUSP-2023-0047en

**Published:** 2023-08-28

**Authors:** Jéssica Azevedo Guardalupe, Ivana Duarte Brum, Débora Francisco do Canto, Kelly Cristina Milioni Telles, Ana Maria Müller de Magalhães, João Lucas Campos de Oliveira

**Affiliations:** 1 Universidade Federal do Rio Grande do Sul, Programa de Pós-graduação em Enfermagem, Porto Alegre, RS, Brazil.

**Keywords:** Personnel Downsizing, Workload, Nursing Staff, Hospital, Nursing Assessment, Personnel Management, Reducción de Personal, Carga de Trabajo, Personal de Enfermería en Hospital, Evaluación en Enfermería, Administración de Personal, Redução de Pessoal, Carga de trabalho, Recursos Humanos de Enfermagem no Hospital, Avaliação em Enfermagem, Gestão de Recursos Humanos

## Abstract

**Objective:**

To compare nursing staff workload and dimensioning measured by two patient classification systems.

**Method:**

Cross-sectional study, developed in a clinical inpatient unit of a large hospital in southern Brazil, between June and August 2022. Included patients (n = 260) were assessed through two different patient classification systems. The dimensioning calculation provided by the standard and descriptive statistics were applied.

**Results:**

Of the total number of patients, 1,248 classifications were performed with each of the classification systems. One of the instruments showed a concentration of demand for minimal care (54.5%) and the other for intermediate care (63.4%). The anticipation of required nursing hours was discrepant (235.58 and 298.16 hours), as well as the projected nursing staff, which was of 53 and 67 workers, respectively.

**Conclusion:**

Measurement of workload and dimensioning were different when using two patient classification systems in the same sample. Additional accuracy studies shall be carried out.

## RESUMO

**Objetivo:**

Comparar a carga de trabalho e o dimensionamento do pessoal de enfermagem mensurados por dois sistemas de classificação de pacientes.

**Método:**

Estudo transversal, desenvolvido em uma unidade de internação clínica de um hospital de grande porte do sul do Brasil, entre junho e agosto de 2022. Os pacientes incluídos (n = 260) foram avaliados por dois diferentes sistemas de classificação de pacientes. Aplicou-se o cálculo de dimensionamento previsto pela norma e estatística descritiva.

**Resultados:**

Sobre o total de pacientes, foram realizadas 1.248 classificações com cada um dos sistemas de classificação. Um dos instrumentos demonstrou concentração de demanda de cuidados mínimos (54,5%) e outro de cuidados intermediários (63,4%). A previsão de horas de enfermagem requeridas foi discrepante (235,58 e 298,16 horas), bem como a projeção do pessoal de enfermagem dimensionado, que foi de 53 e 67 trabalhadores, respectivamente.

**Conclusão:**

A mensuração da carga de trabalho e o dimensionamento foram distintos ao se utilizar dois sistemas de classificação de pacientes numa mesma amostra. Sugere-se que sejam realizados estudos adicionais de acurácia.

## RESUMEN

**Objetivo:**

Comparar la carga de trabajo y el dimensionamiento del personal de enfermería medidod por dos sistemas de clasificación de pacientes.

**Método:**

Estudio transversal, desarrollado en una unidad clínica de hospitalización de un gran hospital en el sur de Brasil, entre junio y agosto de 2022. Los pacientes incluidos (n = 260) fueron evaluados utilizando dos sistemas de clasificación de pacientes diferentes. Se aplicó el cálculo de dimensionamiento proporcionado por la norma y estadística descriptiva.

**Resultados:**

Del total de pacientes se realizaron 1.248 clasificaciones con cada uno de los sistemas de clasificación. Uno de los instrumentos mostró una concentración de la demanda de cuidados mínimos (54,5%) y otro de cuidados intermedios (63,4%). La previsión de horas de enfermería requeridas fue discrepante (235,58 y 298,16 horas), así como la proyección de enfermería dimensionada, que fue de 53 y 67 trabajadores, respectivamente.

**Conclusión:**

La medición y el dimensionamiento de la carga de trabajo fueron diferentes al utilizar dos sistemas de clasificación de pacientes en la misma muestra. Se sugiere que se lleven a cabo estudios de precisión adicionales.

## INTRODUCTION

Human resource management is considered a factor of direct impact on the quality of health services provided to the population^(1)^. In nursing, this is a complex, uninterrupted dynamics that demands a lot of time from area leaders and managers. Precisely, in the management of human resources in nursing, the processes of staff dimensioning, recruitment, selection, performance evaluation, development, and allocation are included^(2)^.

The dimensioning of nursing staff (DNS) aims to predict the number of workers adjusted by category required to meet the needs of assistance directly or indirectly provided to patients^(3)^. The nurse becomes the protagonist of the dimensioning process, as he/she is responsible for choosing the dimensioning and execution method and also for analyzing the results, instrumentalizing the decision-making process in people management^(4)^.

Research on the association of DNS and workload with several variables of interest in the nursing service demonstrates that inadequate dimensioning is related to the known risks to patient safety and also to negative consequences for the patient’s experience and quality of care, increase in adverse events, and damage to the workers’ health^(5,6,7,8)^.

An observational study carried out in 300 hospitals in nine European countries found that the increase in nurses’ workload raised by 7% the probability of an inpatient dying within 30 days of admission^(9)^. In Brazil, a cross-sectional study carried out in a university hospital found a significant association between workload and the function performed, demonstrating that nursing workers had a greater load, as well as a significant relationship between workload and health strain on workers in general^(8)^.

The parameters for the qualitative and quantitative dimensioning of Brazilian nursing professionals are regulated by the Federal Nursing Council (COFEN) through Resolution No. 543/2017, currently in force^(3)^. For dimensioning in the hospital area, especially in Inpatient Units (IU), the use of a validated Patient Classification System (PCS) is recommended^(3)^.

The PCS corresponds to a systematic means of evaluating the patient in terms of some aspects/areas of interest to nursing care and work, attributing a nurse’s judgment on each one of them and, consequently, fitting the patient in a certain gradation/level of complexity^(10–12)^. Through the PCS workload can be measured, since this instrument shows the variation in the average working time. This way, the PCS provides subsidies for the manager to carry out the reallocation of human resources, helping in the justifications for the decision-making processes related to staff adjustment^(11,13)^.

It is considered that, when comparing different PCSs, dissonances and consonances that influence the accuracy of the DNS can be found. It is likely that this difference in results among different PCSs has a direct impact on the forecast of human resources. These considerations are anchored on the already known fact that the PCSs, including those recommended by current regulations^(3)^, are not exactly the same, despite having in common the logic of classifying the patient in a certain stratum of care complexity. Therefore, the gap in knowledge regarding the possible differences visualized by evaluations with different PCSs is a potential guide for scientific, institutional, and even political (re)planning about the procedures governing DNS in the hospital area.

In view of the factors aforementioned, this study is expected to help consolidate better managerial knowledge and seeks to answer the following research question: “Is there a difference in the workload and consequent dimensioning of nursing staff measured by two different patient classification systems?” Therefore, the objective of this study was to compare the workload and dimensioning of the nursing staff measured by two patient classification systems.

## METHOD

### Design of Study

Cross-sectional, descriptive study with a quantitative approach.

### Context

The study was carried out in a medical inpatient unit of a large public teaching hospital in southern Brazil. The unit has 45 beds and provides care in several clinical specialties, being an institutional reference in oncology. All beds are accredited by the Brazilian Public Health System.

### Participants

The study population consisted of all patients admitted to the unit during the data collection period. The composition of the non-probabilistic, consecutive and convenience sample was based on the inclusion of patients with a minimum hospital stay of 24 hours. Patients under 18 years of age who were not accompanied by a family member and/or guardian were excluded, as well as adult patients who were unable to respond to consent (due to clinical and/or psychological reasons) and did not have a companion. Such conditions were checked by a qualified researcher.

### Variables

The study variables were classified into: 1) demographic variables: age and sex; 2) variables related to hospitalization: days of hospitalization and medical specialty responsible for the hospitalization; and, 3) managerial variables: care complexity level measured by two different PCSs and number of beds occupied in the unit.

### Quantitative Variables

Data collected were stored in spreadsheets of the software *Microsoft Office Excel* ^®^ where descriptive statistical analysis was performed. Categorical variables were expressed in measures of percentage proportion and absolute frequency, and quantitative variables were described as mean, standard deviation, median, and interquartile range.

### Bias

Data collection was carried out by a research team consisting of two nurses from the unit, one of them a doctoral student in nursing, and the other a student with an undergraduate research scholarship, both directly supervised by an investigator in the DNS area. The distinct qualification of the collectors was considered a collection bias, which was minimized through training in a pilot study prior to data collection in two stages: before consensus on the items of the two PCSs and after consensus among the evaluators. During each of the steps, 15 patients were evaluated independently by each evaluator. On the whole, 180 pilot classifications were carried out, 90 for each PCS. In the pre-consensus period, the overall agreement within assessments in the PCS by Fugulinet al.^(11)^ was 67% and in the PCS by Perroca^(12)^ was 60%. After consensus, the overall agreement within assessments was 93.3% for both PCS. The few residual discrepancies were dealt with in a team meeting and considered duly resolved.

### Data Source

The PCSs by Fugulin et al.^(11)^ and Perroca^(12)^ were used. These instruments were selected because the PCS by Perroca is currently used by the study hospital and the one by Fugulin is one of the most used to assess adult patients hospitalized in internal medicine units, according to the literature^(2,14)^. In addition, both instruments are recommended by COFEN Resolution No. 543/2017^(3)^. The classification of patients with the two instruments was carried out for a total of 30 days in the cited time frame, as recommended by researchers in this field^(2,13)^. On each day of data collection, hospitalized patients were evaluated according to the two chosen classification systems.

The PCS by Perroca^(12)^ comprises nine areas of nursing care required by hospitalized patients: 1. Planning and coordination of the care process; 2. Investigation and monitoring; 3. Body care and voiding; 4. Skin and mucous care; 5. Nutrition and hydration; 6. Locomotion and activity; 7. Therapy; 8. Emotional support; 9. Health education. Each of the areas can be scored from 1 to 4, with higher scores indicating increased levels of complexity. The level of complexity of patient care is defined by the sum of the points of all indicators. The sum is illustrated in four categories: minimum care from 9 to 12 points; intermediate care from 13 to 18 points; semi-intensive care from 19 to 24 points; and intensive care from 25 to 36 points. The version of the instrument used in this study was adapted by the nurses at the institution where the research was carried out, being duly validated and authorized by the author^(15)^.

The PCS by Fugulin also comprises nine areas of nursing care, namely: 1. Mental status; 2. Oxygenation; 3. Vital signs; 4. Motility; 5. Ambulation; 6. Feeding; Body care; 8. Voiding; 9. Therapy. Each of the areas has a gradation from 4 to 1 and the definition of each category of care determines the patient’s care complexity. The sum of the scores varies from 9 to 36 points, with the ranges defined by five categories: minimum care from 9 to 14 points; intermediate care from 15 to 20 points; high-dependent care from 21 to 26 points; semi-intensive care from 27 to 31 points; and intensive care above 31 points^(11)^.

For sociodemographic, managerial, and clinical variables, a semi-structured questionnaire containing the following variables was applied: number of occupied beds, age, sex, medical specialty responsible for the hospitalization and days of hospitalization. These data were collected *in loco* when visiting patients and consulting medical records.

### Data Collection

Data collection was carried out from June to August 2022, for a total of 30 days – considered a sufficient minimum to perform the DNS^(2)^ – and not uninterrupted and random in this time frame. The interruption in the collection period was elected to provide study feasibility and increase the variability of the clientele evaluated by the PCS.

### Statistical Methods

The method and the dimensioning parameters used on the results of both PCSs were recommended by COFEN Resolution No. 543/2017. Therefore, according to the results of each of the PCSs, the calculations of the staff (*QP*) dimensioned in the unit were performed using the equation: QP = THE X KM. The daily hours of each category/level of care dependence of each PCS were considered as parameters for the calculation of the Total Nursing Hours (*THE*), based on the average number of patients in each stratum/level of each PCS in the 30-day period of clientele assessment.

In this study, a minimum Technical Safety Index (*IST*) of 15% and Marinho’s Constant (*KM*) of 0.2236 were considered, which refer to a weekly workday of 36 hours, 7 days of work a week (non-stop hospital work)^(3)^. The *IST* is the percentage to be added to the number of professionals to ensure coverage of vacations and unpredicted absences. The IST of at least 15% of the total should be added to the established number of professionals, of which 8.3% refer to vacations and 6.7% to unpredicted absences^(3)^.

The nursing staff dimensioned by the results of the PCS by Perroca and Fugulin were proportionally adjusted among nurses and nursing technicians/assistants, using the current normative parameters and considering the category/level of care complexity with the greatest demand for nursing hours^(3)^. Finally, to compare the dimensioned tables with the available/actual one at the inpatient unit, data provided by the hospital’s Personnel Management Coordination were used.

### Ethical Aspects

The project that fostered this study was submitted and approved by the research ethics committee, under opinion no. 4.932.314 of 2021, in accordance with Resolution 466/12 of the National Health Council^(16)^, using an instrument of consent.

## RESULTS

A total of 260 patients was included. Of these, 1,248 classifications were performed with each PCS among the patients. Therefore, the sum within the two instruments totaled 2,496 evaluations.

Among the patients, there was a slight predominance of females (52.3%), with a mean age of 56.1 ± 17.1 years, mostly hospitalized by the specialty of Oncology (23.5%). Other information about the clinical and demographic profile of patient sample is presented in Table 1.

**Table 1:** Clinical and demographic profile of the sample of hospitalized patients – Porto Alegre, RS, Brazil, 2022.

Variables	(n = 260)
Sex (Female)*	136 (52.3)
Age (years)^π^	56.1 ± 17.1
Medical specialty*	
Oncology	61 (23.5)
Gastroenterology	34 (13.1)
Cardiology	33 (12.7)
Internal medicine	30 (11.5)
Hematology	25 (9.6)
Nephrology	19 (7.3)
Pulmonology	12 (4.6)
Neurology	11 (4.2)
Infectology and Rheumatology	8 (3.1)
Endocrinology and Pain Management	7 (2.7)
Vascular surgery	2 (0.8)
Coloproctology, Dermatology and Geriatrics	1 (0.4)
**Days of hospitalization** ^π^	8.7 ± 10.8
**Unit Occupancy (Beds)** ^π^	43.6 ± 1.8

Source: survey data.

*Variables expressed in absolute numbers and (%);

^π^mean ± standard deviation.

Table 2 presents the distribution of dependence level on nursing care, by PCS. Regarding the classification score of the Fugulin instrument, the minimum care level stood out (54.5%), and PCS by Perroca showed a predominance of intermediate care (63.4%).

**Table 2:** Distribution of dependence level on nursing care, by Patient Classification System (PCS) within evaluations – Porto Alegre, RS, Brazil, 2022 (n = 1,248).

Dependency level on nursing care*	PCS by fugulin (n = 1,248)	PCS by perroca (n = 1,248)	General sample (n = 2,496)
Minimum Care	680 (54.5)	82 (6.6)	762 (30.5)
Intermediate Care	333 (26.7)	792 (63.4)	1,125 (45.1)
High Dependence Care	217 (17.4)	–	217 (8.7)
Semi-Intensive Care	18 (36.7)	358 (28.7)	376 (15.1)
Intensive care	–	16 (1.3)	16 (0.6)

Source: survey data.

*Variables expressed in absolute numbers and (%).

Table 3 demonstrates the findings regarding the required daily nursing hours, by level of care dependence, between the two PCSs.

**Figure 1:**
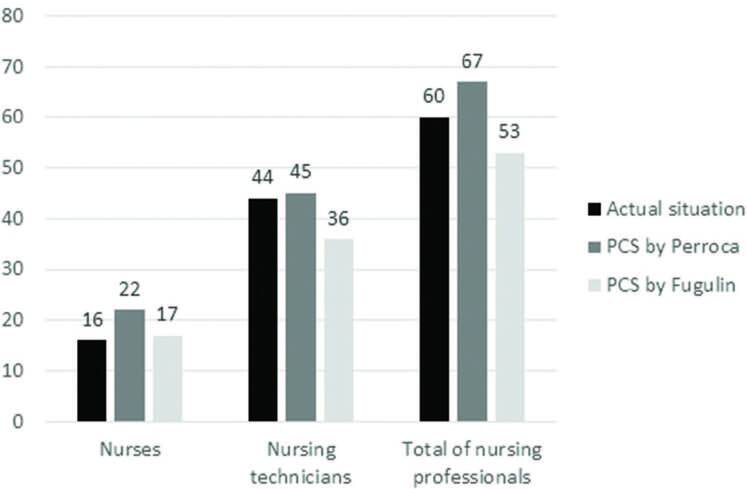
Comparison of the staff dimensioning projected by the PCSs by Perroca and Fugulin with the actual/available staff in the hospitalization unit – Porto Alegre, RS, Brazil, 2022.

With the number of required nursing hours, the nursing staff were properly dimensioned according to the two PCS. In this regard, Figure 1 demonstrates that the staff projected by the Pcs by Perroca showed a greater discrepancy compared to the actual/available staff.

**Table 3:** Nursing hours required by care dependence level and Patient Classification System (PCS) – Porto Alegre, RS, Brazil, 2022.

	PCS by Fugulin		PCS by Perroca	
	Average number of patients	Hours required	Average number of patients	Hours required
**Dependence Level on Nursing Care**				
Minimum Care	22.67	90.68	2.73	10.92
Intermediate Care	11.1	66.6	26.4	158.4
High-Dependence Care	7.23	72.3	–	–
Semi-Intensive Care	0.6	6	11.93	119.3
Intensive care	0	0	0.53	9.54
**Total Nursing Hours**	–	235.58	–	298.16

Source: survey data.

## DISCUSSION

The comparison between the dimensioning of nursing staff, according to each PCS, showed a significant difference between the results provided by the instruments. A study carried out in a philanthropic trauma hospital in Rio Grande do Sul used the instruments by Perroca and Fugulin to classify hospitalized patients. The average score of the 157 patients evaluated was 18.74 in Fugulin’s, whose care complexity was classified as intermediate care and, in Perroca’s, the patients evaluated had a mean score of 27.04, classified as intermediate care^(17)^. In the present study, similar scores were found in PCS by Perroca, with an average score of 26.4 classified as intermediate care; however, in PCS by Fugulin, the mean score found was 22.67 with the minimal care classification.

Regarding Perroca’s classification system, 30% of patients were classified as semi-intensive or intensive care. Intensive care is not expected in a non-critical inpatient unit. However, the clinical profile of the most prevalent unit is cancer patients (23.5%), who require more complex care. A study comprising patients with breast cancer in a clinical oncology unit in Rio de Janeiro found a predominance of semi-intensive care (36.1%) and intensive care (36.1%) when using the PCS by Perroca^(18)^.

In the classifications with the PCS by Fugulin, it was evidenced that more than half of the patients were classified as requiring minimal care, and no patient was classified as requiring intensive care. Research carried out in an internal medicine unit of a university hospital also found a predominance of patients classified as minimal care (35.8%) when using the same PCS^(19)^. This, together with the previously discussed result, is a finding that deserves to be critically analyzed by managers and decision makers, as it attests that the nursing staff forecast is clearly influenced by the selection of the workload measurement instrument, which in the case of hospitalization, is centered on patient classification^(10)^. This does not mean that this study indicates that a PCS is better or more accurate than the other, even because the empirical study results do not account for statements like these, but rather, that there is a difference in the evaluation of patients and consequent staff projection through the application of different PCSs, even if both are validated and recommended.

COFEN Resolution No. 543/2017 regulates the parameters for dimensioning the number of professionals. For dimensioning in inpatient units, the aforementioned document suggests five PCSs as methodological instruments to support the dimensioning calculation, among them, three related to medical-surgical patients, one aimed at pediatric clients, and another for psychiatry^(3)^. Despite the resolution not distinguishing the instruments, in this study it was clearly possible to observe differences in results between them.

The PCS by Fugulin is a more objective instrument in the description of its indicators within each area. In addition, it does not consider the time demand and impact on the workload of the systematization of nursing care (*SAE*), of the emotional support, and of health education. All these activities demand time from the nursing team. The Nursing Process, for example, is an essential activity performed by nurses to organize care. Considering that the PCS by Perroca covers these three areas, it is possible that this is related to the greater number of patients classified as intermediate care compared to the predominant minimum care identified by the PCS by Fugulin.

In addition, the PCS by Fugulin uses the category of high-dependence care patients, which is not used by the PCS by Perroca. Chronic patients, including palliative care patients, who are stable from a clinical point of view but totally dependent on nursing actions to meet basic human needs, are considered highly dependent^(3)^. In this study, 17.4% of the sample was classified as highly dependent.

In view of the non-existence of the high-dependence care level, it is possible that in the PCS by Perroca, patients who qualify as high dependent are directly classified as semi-intensive. Therefore, it is assumed that the PCS by Perroca tends to assess some items more rigorously compared to the PCS by Fugulin, reflecting in higher levels of dependence. COFEN considers that “high dependence” and “semi-intensive” levels of care equally demand 10 daily nursing hours per patient. However, for the percentage distribution of the total number of nursing professionals, a minimum proportion of 36% of nurses and the other technicians and/or assistants for high dependence care and 42% of nurses and the other technicians for semi-intensive care is indicated^(3)^. Therefore, the resolution implies that the existence of different dependence levels between the PCSs can lead to differences during the dimensioning process, mainly in the distribution within professional categories.

In this study, the staff dimensioned according to the current specific standardization was 17 nurses and 36 technicians/nursing assistants, according to Fugulin’s classification. The staff dimensioned by Perroca’s PCS was 22 nurses and 45 technicians/nursing assistants. The unit’s real staff consisted of 16 nurses and 44 nursing technicians/assistants. It can be seen that a surplus of the actual staff in relation to the staff dimensioned by Fugulin’s PCS was predicted, with 8 “excess” nursing technicians and a deficit of 1 nurse, which resulted in 7 more nursing professionals. In relation to the staff dimensioned by Perroca, a deficit of professionals was found when compared to the real staff, with minus 6 nurses and minus 1 nursing technician/assistant, totaling a deficit of 7 workers. This finding is, perhaps, the greatest implication with the potential for transferring knowledge arising from this study, because it can mark out in a more rational or conscious way the selection of a PCS by nursing managers.

An oversized team is directly related to high institutional costs. However, a deficit in staff dimensioning exposes workers to work overloads. The shortage of nursing staff is related to impairment of quality and quantity of care provided, and enhances stress, exhaustion, and conflict among professionals^(20)^.

A study carried out in four public hospitals in Iran with the participation of 616 nurses found a significant association between work overload and all types of occupational accidents evaluated^(21)^. Another problem is that the lack of nurses makes nursing technicians and assistants help, or even become responsible, for activities that are not within their professional scope^(20)^.

Discussions about the applicability of PCS have been increasing. There are several nursing activities that are not sensitive to the application of these instruments, which can result in an underestimated workload^(10)^. However, the objective of these instruments is to contain the dimensions of care that most impact the workload of the nursing team, as a list that includes all the activities performed by the nursing staff is unfeasible^(12)^.

A multicenter study carried out in the Netherlands found that six items determine the adequacy of the staff, with the nurse being able to: complete care activities, provide care according to protocol or guideline, prepare for discharge with the patient and family, educate the patient, be able to take breaks, and guide nursing students^(22)^. Therefore, it is necessary to reflect that the study described here was carried out in a university hospital that has several students, from undergraduate and graduate courses, supervised by nurses, an activity not contemplated in any of the PCSs used.

Finally, the characterization of the demographic data of hospitalized patients showed a predominance of female patients and a mean age of 56 ± 17.1 years, data related to a recent study carried out in Ceará that investigated hospitalized oncology clients^(23)^. The average length of hospital stay can be considered high according to the recommendation of general average length of stay for large hospitals of 4-5 days^(24)^; however, other studies point to similar results^(25)^. This point can be explained due to the fact that clinical patients have an epidemiological profile of chronic diseases with recurrent exacerbations, resulting in the need to seek care at higher levels of complexity and consequent prolonged hospitalization time^(14)^.

The main limitation of this study was the lack of data on the absences and presences of workers in the inpatient unit in question, which could result in the redefinition of the *IST* used. Despite not being part of the scope of the study, other limitations include not verifying the prefered PCS to be used and the time spent evaluating each of the instruments, which, in addition to the analysis of the accuracy of these and any other PCSs, shall be points considered in future studies so that more assertive decisions regarding the planning of nursing staff can be envisaged.

This is one of the few studies in Brazil comparing patient classification systems and possibly the first to relate such a comparison to the dimensioning of nursing staff. Moreover, one of the main contributions of the study is evidence that assessments carried out by different PCSs differ in several aspects, which can have a direct impact on the dimensioned nursing staff. That is, the selection of the PCS is a crucial decision during the dimensioning process. Hopefully, the results of the study will serve as an incentive for nurses, researchers, and managers to carry out new studies on the subject and also be precursors for the review of procedures related to DNS in Brazil.

## CONCLUSION

The measurement of the workload and the dimensioning of the nursing staff were clearly different when using two different PCSs in the same patient sample, which highlights the importance of more advanced reflections on the applicability of PCSs and their limitations. The nursing activities evaluated in each of the instruments differ from each other, as well as the final classification categories of the patients; however, more than that, nursing staff projections for the same unit (in theory, the same workload demand) are very different. This directly influences the budget distribution of the nursing service, and presupposes the possibility of incurring staff under- or overestimation, which is considerably relevant.

It was observed that one of the PCSs does not consider the time demand and the impact on the workload arising from the nursing process, the emotional support provided to the patient/family, and health education. The PCS by Perroca tended towards a more rigorous determination of the nursing workload and, consequently, projected a greater volume of required working hours and dimensioned professionals.

The findings do not indicate that one PCS is better than the other. Therefore, it is suggested that additional accuracy studies be carried out and it is hoped that these data will be considered in institutional and political-professional decisions on hospital nursing staff planning.
